# Olfactory adaptation: recordings from the human olfactory epithelium

**DOI:** 10.1007/s00405-021-07170-0

**Published:** 2021-12-18

**Authors:** Coralie Mignot, Anica Schunke, Charlotte Sinding, Thomas Hummel

**Affiliations:** 1 TU Dresden, Smell and Taste Clinic, Department of Otorhinolaryngology, House 5, Basement, Fetscherstraße 74, 01307 Dresden, Germany; 2 Department French National Institute for Agricultural Research, Centre des Sciences du Goût et de l’Alimentation (CSGA), Dijon, France

**Keywords:** Adaptation, Habituation, Electro-olfactogram, EOG, Intensity, Repeated stimulations

## Abstract

**Purpose:**

Olfactory adaptation is a peripheral (at the epithelium level) or a central (at the brain level) mechanism resulting from repeated or prolonged odorous exposure that can induce a perceptual decrease. The aim of this study was to assess whether a peripheral adaptation occurs when an odor is repeated ten times. Moreover, the specificity of the peripheral adaptation to the nature of the odorant was investigated.

**Methods:**

Four odorants (eugenol, manzanate, ISO E Super and phenylethanol) were presented using precisely controlled air-dilution olfactometry. They differed in terms of their physicochemical properties. Electrophysiological recordings were made at the level of the olfactory mucosa, the so-called electro-olfactogram (EOG). Thirty-five right-handed participants were recruited.

**Results:**

Sixty-nine percent of the participants presented at least one EOG, whatever the odor condition. The EOG amplitude did not significantly decrease over 10 repeated exposures to any odorant. The intensity ratings tended to decrease over stimulations for manzanate, PEA, and eugenol. No correlation was found between the mean EOG amplitudes and the mean intensity ratings. However, the presence of EOG amplitude decreases over stimulations for few subjects suggests that peripheral adaptation might exist.

**Conclusion:**

Overall, our results did not establish a clear peripheral adaptation measured with EOG but indicate the eventuality of such an effect.

**Supplementary Information:**

The online version contains supplementary material available at 10.1007/s00405-021-07170-0.

## Introduction

We are surrounded by hundreds of cues which attentional priority needs to be constantly addressed to react quickly in a changing environment. Thus, some mechanisms are needed to define the priority of processing the incoming stimulations. For this purpose, habituation and adaptation are necessary to sort out what is neutral/uninformative from what constitutes a meaningful stimulation in a specific context; this is particularly true in olfaction which allows detecting danger [1]. The olfactory adaptation is a peripheral or a central mechanism resulting from a repeated or prolonged odorous exposure that induces a decrease in responses or behaviors. This consequence is called habituation, which may be translated into a progressive decrease in some perceptual components [2] such as a decrease in intensity [3, 4], a change in hedonicity that tends toward neutrality [5], etc. If these filters did not exist, the brain would be overwhelmed with information very quickly.

Adaptation is due to several mechanisms either at the level of olfactory receptors (peripheral adaptation) or at the neuron’s level within the olfactory epithelium (peripheral adaptation) or within the different brain areas included in the olfactory cortex (central adaptation). There are similar adaptation effects in other senses, combining peripheral and central adaptation. In vision, the photoreceptors already take over the changes in average brightness or color, while some other features such as direction of motion of light are modulated in a central way [6]. The retinal layer in the eye is also prone to light adaptation while contrast adaptation happens both at the retina level and in the cortex [7]. Concerning audition, it has been shown that electrical pulses at the level of the auditory nerve elicit adaptation [8], but that plasticity also happens in multiple locations in the nervous central system in order to maintain perceptual stability in sound levels, speech recognition, and other features of the auditory sense [9]. Adaptation to taste happens both at a peripheral level and centrally [10, 11]. In the somatosensory system, vibrotactile habituation occurs with skin elasticity changes, nerve fibers desensitization and central adaptation [12].

The debate of which of the peripheral or central adaptation contributes mostly to olfactory habituation is still open, especially the possible existence of a central/peripheral feedback loop [13]. Some studies have been made in animals to assess the mechanisms of peripheral adaptation, cf. review [14]. However, this question has been sparsely investigated in humans, especially in the context of many repeated exposures.

The first reason is that it is very difficult to record olfactory receptor responses of the human olfactory epithelium. An electrode has to be positioned as gently as possible on the olfactory mucosa to record the electrical potential of discharge of a group of olfactory sensory neurons (OSNs). The technique is called the electro-olfactogram (EOG) [15]. Osterhammel and colleagues were the first to measure EOG in humans [16]. The receptors responsible for olfaction are located on the OSNs. When an odorant binds to the receptor, usually connected to a G protein, this initiates a metabolic cascade resulting in a depolarization of the membrane. The signal measured with EOG is the sum of all of these OSN generator potentials. The EOG shape is generally represented by a fast and short increase in potential (positive transient potential), followed by a slow decrease (negative transient potential) [17, 18]. The cause of the positive voltage transient is still debated but is unrelated to the olfactory activation [18], while the negative component is due to the depolarization of the OSNs [19].

To our knowledge, only two studies investigated human peripheral adaptation using EOG. These works examined whether two olfactory stimuli separated by various inter-stimulus intervals would exhibit different EOGs. The results showed a slight decrease in amplitude from the first stimulation to the second one which was almost as large as the response to the first stimulus [20, 21]. However, longer stimulation is usually necessary to see habituation response. Therefore, we tackled this question while repeating 10 times the stimulations.

Some parameters are known to affect habituation, such as the number of stimulus repetitions, the duration of exposure, the initial intensity and pleasantness of the odor [2, 5, 13, 22], the trigeminality, the vapor pressure, the number of double bounds, and the molecular weight of the odorants [23]. According to the combination of these parameters, some odorants can be classified as low habituation inducers, while some others are high habituation inducers [23]. Again, this diversity in adaptation leading to habituation has never been studied systematically at a peripheral level in humans.

This study aimed to assess whether peripheral adaptation occurs when a specific odor is repeated ten times. Moreover, the specificity of the peripheral adaptation to the nature of the odorant was also investigated. To this end, the EOGs in response to four different odors were recorded using a repeated stimulation paradigm.

## Materials and methods

### Participants

Thirty-five right-handed participants were recruited for this experiment (mean age = 25 ± 3.5 years, range 18–35 years, 17 men and 18 women). The exclusion criteria were the following: pregnancy, major chronic disease, olfactory loss, nasal surgery, asthma, attentional dysfunction, and smoking. Nine participants had seasonal allergies to allergens occurring in summer, which were not present during the experimental period (autumn/winter); thus, they were included in the study. All participants provided written informed consent. The data were collected in accordance with the declaration of Helsinki related to human research, and the protocol was approved by the Ethics Committee of the Faculty of Medicine at the “Technische Universität Dresden” (GVOEK) under the application number EK 95,032,014. For their participation, the subjects received moderate financial compensation after the experiment.

### Odorants

Four odorants (Givaudan Ltd, Ashforf, UK) were used: eugenol (1-Hydroxy-2-Methoxy-4-Allyl-Benzol), ISO E Super (7-Acetyl- 1,2,3,4,5,6,7,8-Octahydro-1,1,6,7-Tetramethyl-Naphthalene), manzanate (Ethyl 2-Methyl-Pentanoat) and PEA (2-Phenylethanol). They were selected based on previous work [23] showing that odorants elicit different short-term habituation patterns depending on their physicochemical properties. Thus, manzanate, eugenol, and ISO E Super represented three different types of short-term habituation (respectively, low, middle and high). PEA was used as a search stimulus to determine the best position of the EOG electrode on the olfactory epithelium, as PEA is known to produce only activation of the olfactory nerve with little or no trigeminal activation [24].

The odorants were presented using a computer-controlled olfactometer (OM6b; Burghart-Messtechnik, Wedel, Germany) at a humidity rate of 80% and a temperature of 36 °C to resemble nasal conditions. First, the liquid odorants were placed in the modules of the olfactometer at a neat concentration, except the manzanate that was diluted at 0.125% in propylene glycol. In a further step, the odorous airflows were mixed with an odorless airflow (total airflow of 6.4L/min), the final concentrations were the following: EUG 27.656%, ISO E 35.625%, MAN 0.023% and PEA 37.188%. These concentrations were chosen to ensure an identical intensity but at a minimum level in order to facilitate habituation. The odorants were delivered in blocks of 10 repetitions, with a duration of 1 s and an inter-stimulus interval of 4 s. The steepness of the stimulus was < 30 ms. No significant difference was found between all odors in terms of intensity of the first stimulation out of ten (repeated measures ANOVA df = 3, *F* = 1.32, *p* = 0.27).

### Procedure

Two sessions were performed: in the first one, the psychophysical testing was done, an intensity rating procedure was conducted, and the participant was trained to be used to the velopharyngeal closure procedure, the setup and the placement of the electrode. The velopharyngeal closure is a method that avoids the flow of respiratory air in the nose while breathing through the mouth. The electrophysiological measurement was conducted in the second session. These appointments were separated in average by 11 days (minimum 7 days, maximum 20 days).

The experiment took place in an air-conditioned room set at a temperature of 20–22 °C. The participants were submitted to nasal endoscopy to ensure the absence of any anatomical abnormality or major nasal pathology. Then, the EOG electrode was positioned. In the first session, they received 10 repetitions of odorous air puff for each odor and were asked to rate the intensity of each puff by the means of a Visual Analog Scale (VAS). This scale ranged from 0 (no sensation) to +  +  + (very high intensity) and appeared 1 s after the odor presentation on a screen placed at 2 m from the subject. In the second session, they received passively the stimulations while they performed a tracking task to maintain their attention throughout the experiment. All participants received first a CO_2_ stimulation (concentration 50%, duration 500 ms) that produces a typical easily recognizable potential that validates the positioning of the electrode on the nasal mucosa. Each participant then received the 4 odorants in a counterbalanced order.

### Psychophysics

The general olfactory ability of the participant was checked at the first session using the 16-item identification task from the Sniffin’ Sticks olfactory test battery [25, 26]. The participants were included in the study if they were able to identify 12 out of the 16 odors of the test. Based on this criterion, all of them were included.

The trigeminal ability of the participants was checked using the lateralization task described elsewhere [27, 28]. In this task, two identical airflows were applied to both nostrils using a handheld “squeezing device” which releases the same amount of air simultaneously to the left and right nostrils. One side received the target odorant, while the other side received odorless air. The sides of the odorant stimulation were changed in pseudo-randomized order. If the odorant has a trigeminal component, the success rate in detecting the stimulated nostril increases significantly. Participants had to perform the localization task 20 times. When comparing the success rates using paired sample *t* tests, none of the odorants (EUG, ISO E, MAN, PEA) could be better localized than the others (*p* > 0.1).

### Electro-olfactogram recording

A tubular electrode filled with Ringer-agar (1%) containing a silver chloride wire (diameter of the wire of 0.3 mm, inner diameter of the tubing of 0.4 mm, outer diameter of the electrode of 0.8 mm) that recorded the EOG while Ag/AgCl electrodes were used as reference (two on the earlobes) and to identify vertical eye-blinks in the signal (two above the lateral extremity of the eyebrows). The EOG electrode placement was controlled using a rigid endoscope with a 30° optic (Karl Storz, Tuttlingen, Germany). The nasal tubing of the olfactometer was inserted in the nasal cavity close to the outer part to let enough space for the EOG electrode to reach the olfactory epithelium.

The recording was performed with a bioamplifier (PowerLab 26 T, AD Instruments, Oxford, United Kingdom) and its associated software LabChart (AD Instruments, Oxford, United Kingdom). The sampling rate was 1 kHz and a notch filter was applied at 50 Hz. In case the signal exceeded 20 mV, the participant was invited to change his position to sit more comfortably and to practice the velopharyngeal closure.

### Electro-olfactogram analysis

A high-pass filter was applied at 0.1 Hz and a notch filter at 50 Hz. The signal was verified for each participant, odor and stimulus. The presence and amplitude of the potential EOG were assessed with the software LabChart (AD Instruments, Oxford, United Kingdom). The presence of the EOG was taken into account when: (1) an obvious deviation from the baseline was observed at least for the first EOG, (2) the shape of the EOG corresponded to a small positivity (P1) followed by a large negativity (N1) or only a large negativity, (3) the onset of the EOG occurred less than 0.25 s after the odor trigger, and (4) no eye blink occurred at the same time.

### Data analyses

A Linear Mixed Model was used to add the participants as a random effect. The N1 amplitudes and intensity ratings were tested, the ten triggers were used as fixed effect. A *p* < 0.05 was considered as significant while a *p* ≤ 0.10 is mentioned as a tendency. Analyzes were made using JASP [JASP Team (2020). JASP (Version 0.14.1) (Computer software)] and SPSS (Statistical Packages for Social Sciences, Version 23.0, SPSS Inc., Chicago, USA) software.

## Results

### Intensity ratings

**Fig. 1:**
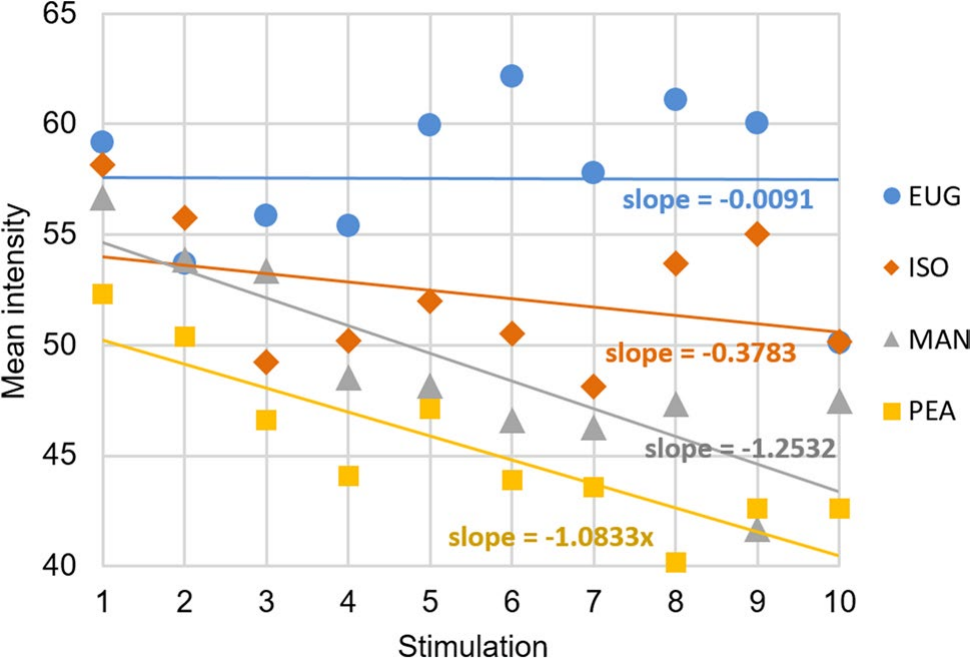
Mean intensity ratings over repeated stimulations for EUG, ISO E, MAN and PEA. *EUG* eugenol, *ISO* Iso E Super, *MAN* manzanate, *PEA* phenyl–ethyl alcohol. The intensity scale was reframed from 40 to 65 to focus on the slopes

MAN and PEA individual intensity ratings decreased over stimulation (MAN df = 9, *F* = 2.799, *p* = 0.004; PEA df = 9, *F* = 1.957, *p* = 0.044), while EUG ones tended to decrease (df = 9, *F* = 1.823, *p* = 0.063) and no significant decrease was found for ISO E (df = 9, *F* = 1.470, *p* = 0.158), see Fig. 1. For individual intensity ratings, means and confidence intervals, see Supplementary data.

### Descriptive EOG

Sixty-nine percent of the participants presented at least one EOG, whatever the odor condition (40% had at least one EOG with EUG, 43% with ISO E, 46% with MAN and 34% with PEA). Among the participants who had EOGs, only 43% of those stimulated with EUG presented 4 or more EOGs in a row, 27% for ISO E, 31% for MAN and 50% for PEA (for the complete distribution, see Table 1).

**Table 1:** Number of subjects presenting electro-olfactograms (EOG) for each odor

N. of EOGs	Number of subjects having EOGs with			
	EUG	ISO	MAN	PEA
10	3	2	3	3
9	0	2	1	1
8	2	0	0	1
7	0	0	0	0
6	0	0	0	0
5	0	0	1	1
4	1	0	0	0
3	1	0	4	0
2	2	4	3	4
1	5	7	4	2
0	21	20	19	23

*EUG* eugenol, *ISO* Iso E Super, *MAN* manzanate, *PEA* phenylethyl alcohol. As an example, 3 subjects presented 10 EOGs when stimulated with EUG, no subject presented 9 EOGs when stimulated with EUG

**Fig. 2:**
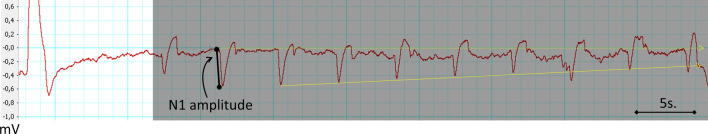
Typical EOG responses over 10 stimulations. The figure represents the signal of a single subject exposed to EUG. The EOG is usually described as a small positivity followed by a large negativity, or sometimes just a large negativity. The amplitude is expressed as mV. The gray part highlights the period during which the stimulations were delivered. A decrease of N1 amplitude over stimulations can be observed here. The positivity at the beginning of the recordings represents an eye blink

Among the 11 recordings with 10 clear EOGs in a row, 5 subjects presented a decrease in EOG N1 amplitudes over the 10 stimulations for some of the odors (as can be seen on the Fig. 2).

### EOG, N1 amplitudes

**Fig. 3:**
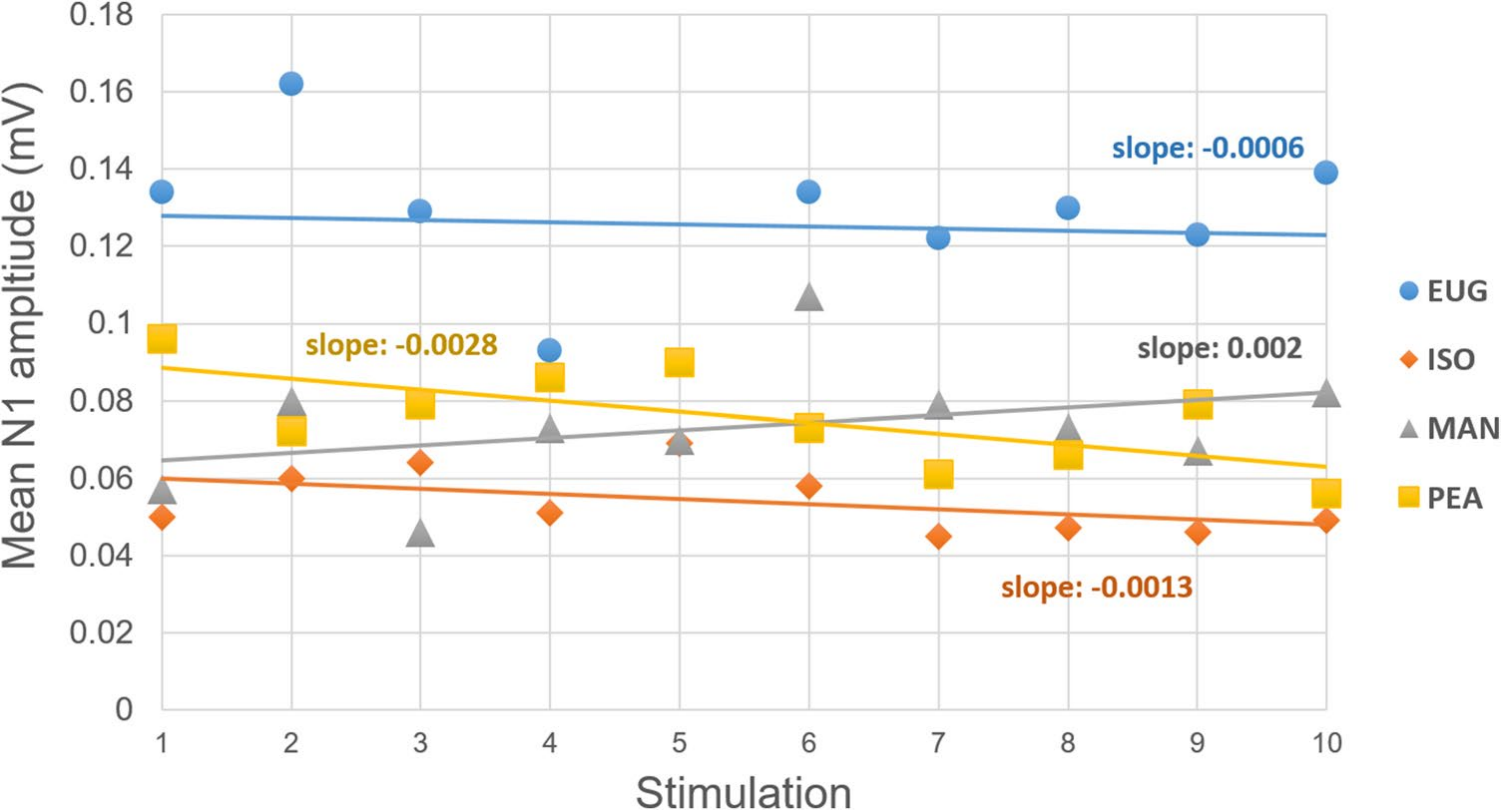
Mean N1 amplitudes over stimulations for EUG, ISO E, MAN and PEA. *EUG* eugenol, *ISO* Iso E Super, *MAN* manzanate, *PEA* phenyl–ethyl alcohol

No significant decrease of N1 amplitudes was found for any of the odorants (*p* > 0.1) (slopes for EUG − 0.0006, ISO E − 0.0013, PEA − 0.0028, EUG: 0.002), see Fig. 3. For individual N1 amplitudes, means and confidence intervals, see Supplementary data.

### Correlation between mean N1 amplitudes and mean intensity ratings

The mean N1 amplitudes and the mean intensity ratings did not correlate for any of the odors.

### Lateralization test

The lateralization task conducted with EUG, MAN, ISO E and PEA showed that participants were not better in localizing EUG, MAN and ISO E compared to PEA (Paired *t* test, *p* > 0.1; except for PEA-ISO for which a Wilcoxon singed-rank test was performed, *p* > 0.1).

## Discussion

In this work, the EOG N1 amplitude did not significantly change over 10 repeated exposures to eugenol, Iso E Super, manzanate or PEA, for most of the participants. However, for few participants, we found a decrease in EOG N1 amplitudes over 10 repetitions. The intensity ratings decreased over stimulations for manzanate and PEA, and tended to decrease for eugenol. No correlation was found between the mean EOG N1 amplitudes and the mean intensity ratings.

It has been shown that short-term peripheral adaptation occurs with pure trigeminal stimuli [29]. This was assessed with the so-called Negative Mucosa Potential (NMP) [30]. The NMP reflects activation of nasal epithelial nociceptors, whose signal can become mixed up with EOG recordings. Here, the lateralization task conducted with the four odors showed that participants were not better in localizing EUG, MAN and ISO E compared to PEA. The presently used concentrations were, in fact, selected to be equal in intensity when applied as single stimuli, and were administered at a low intensity to maximize a potential habituation/adaptation effect [31]. This argues against the idea that the responses recorded in the present study could have been contaminated to a large degree by trigeminal activation.

On the other hand, pure odorant repeated (pairwise) stimulations are known to elicit a clear decrease in intensity but a very slight decrease in EOG amplitudes [20]. Although no significant decrease in N1 amplitude could be found, the previous statement is consistent with the MAN and PEA patterns and to a lesser degree with EUG pattern.

Sinding et al. [23] showed that short-term habituation (reduced intensity over prolonged odor exposure) depends on the trigeminality (the less trigeminal, the more short-term habituation) but also on other physicochemical properties including vapor pressure, molecular weight, or number of double bonds. To what extent this could be the case for short-term peripheral adaptation remains unknown.

According to Sinding et al.’s work, MAN should elicit low short-term habituation, middle habituation for EUG and high habituation for ISO E and PEA, which is not consistent with the present work. In fact, we noted an intensity decrease for MAN, PEA and to a lesser extent, EUG exposures and no such phenomenon for ISO E. However, the procedures of the previous and the present experiments were not identical. The intensity estimates were collected over continuous 120-s odorant exposure in Sinding et al.’s study, while being collected over ten repeated stimulations separated by 5-s intervals with odorless air in the present investigation. In addition, odors were used at different concentrations. The concentration of an odor can explain partly its rate of habituation: in general (but not always) the less intense the stimulus, the more pronounced the habituation [2, 13]. In our case, MAN concentration was almost 74 times less concentrated than in Sinding’s study. Thus, it can explain why MAN elicits low habituation in Sinding’s study and high habituation in the present investigation. For EUG, our concentration was 2.7 times higher than in Sinding’s work; thus, our habituation is almost inexistent while it elicited middle habituation in her work. For ISO E Super, our concentration was 5.2 times higher than in Sinding’s study, which could explain why in their work they found high habituation to ISO E Super, while in ours the habituation was low/middle. Overall, this do not compromise the validity of this study because we can refer on our own subjective intensity results to compare with the EOG results.

Regarding the absence of a significant decrease in EOG amplitudes in this work, one can assume that habituation is more related to central than peripheral adaptation. In rodents, the firing rate of neurons in the hippocampus of rats is different depending on the length of inter-stimulus intervals [32], which means that the hippocampus could track the dynamics of odor exposure and adapt itself in consequence for short-term habituation. In humans, this structure seems to be also involved in short-term habituation, as hippocampus has been shown to interact with the primary olfactory cortex and the anterior insula when there is a prolonged exposure to an odor [33] and their respective BOLD signal decreases with longer durations of odor exposure. The level of activation of the primary olfactory cortex could also reflect the level of habituation [34]. Moreover, the amplitude of event-related potentials of trigeminal and olfactory stimuli after a long period of exposure decreased, reflecting central adaptation [35]. In a general manner, habituation has to do with memory and attention. On the attentional point of view, it has been shown for an intermittent exposure to an odor that its perceived intensity can be manipulated by a prior description [36]. Indeed, when describing the odor as “hazardous” (in opposition to “healthy”), habituation to this odor was slower, highlighting the cognitive aspect of such a mechanism. Finally, it has been shown that older and younger subjects show the same long-term habituation pattern although their sensitivity to odors was different [37]. This discrepancy might reflect central adaptation more than peripheral adaptation. Dalton and colleagues earlier shown the same pattern and are in favor of central adaptation [3]. Another argument in this direction is the lack of amplitude decrease in EOG measures (peripheral processing) but the clear amplitude decrease in central brain processing measured with event-related potentials during a pairwise olfactory stimulation procedure described in previous work [17, 21, 38]. No correlation was found between the mean N1 amplitudes and the mean intensity ratings.

However, for a few individuals expressing ten EOGs in a row, a decrease of N1 amplitudes across stimulations could be observed, which justifies the interest of assessing peripheral adaptation with EOG recordings. Thus, the sample size limited our interpretation here: the results suggest that with more subjects and signals it may be that a decrease in EOG amplitudes could be found. It is also not clear how many stimuli repetitions are needed to report an effect. Hence, while our work does not allow to clearly establish whether peripheral adaptation occurs or whether physicochemical properties influence short-term adaptation, the presence of EOG amplitude decreases over stimulations for few subjects suggest that peripheral adaptation might exist.

## Conclusions

This work constitutes the first study using ten stimulations in a row to investigate peripheral adaptation with EOG recordings. Overall, our results did not establish a clear peripheral adaptation measured with EOG but indicate the eventuality of such an effect.

## Supplementary Information

Below is the link to the electronic supplementary material.

Supplementary file1 (PDF 350 KB)

## Data Availability

We will provide data upon request.
