# Supplementary material for: Olfactory adaptation: recordings from the human olfactory epithelium
Source: Eur Arch Otorhinolaryngol. 2021 Dec 18;279(7):3503–10. doi: 10.1007/s00405-021-07170-0 (PMC9130158; doi:10.1007/s00405-021-07170-0)
Supplement: Supplementary file 1 — Supplementary file1 (PDF 350 KB) [file 405_2021_7170_MOESM1_ESM.pdf]

**Olfactory adaptation: recordings from the human olfactory epithelium**

European Archives of Oto-Rhino-Laryngology

Coralie Mignot[a], Anica Schunke[a], Charlotte Sinding[b], Thomas Hummel[a]

[a]*Smell & Taste Clinic Department of Otorhinolaryngology, TU Dresden, Germany*

[b]*Department French National Institute for Agricultural Research, Centre des Sciences du Goût et de l'Alimentation (CSGA),Dijon, France*

**Correspondance to be sent to:** Coralie Mignot: coraliemignot.pro@gmail.com; Smell & Taste Clinic Department of Otorhinolaryngology, TU Dresden, House 5, Basement, Fetscherstraße 74, 01307 Dresden, Germany.

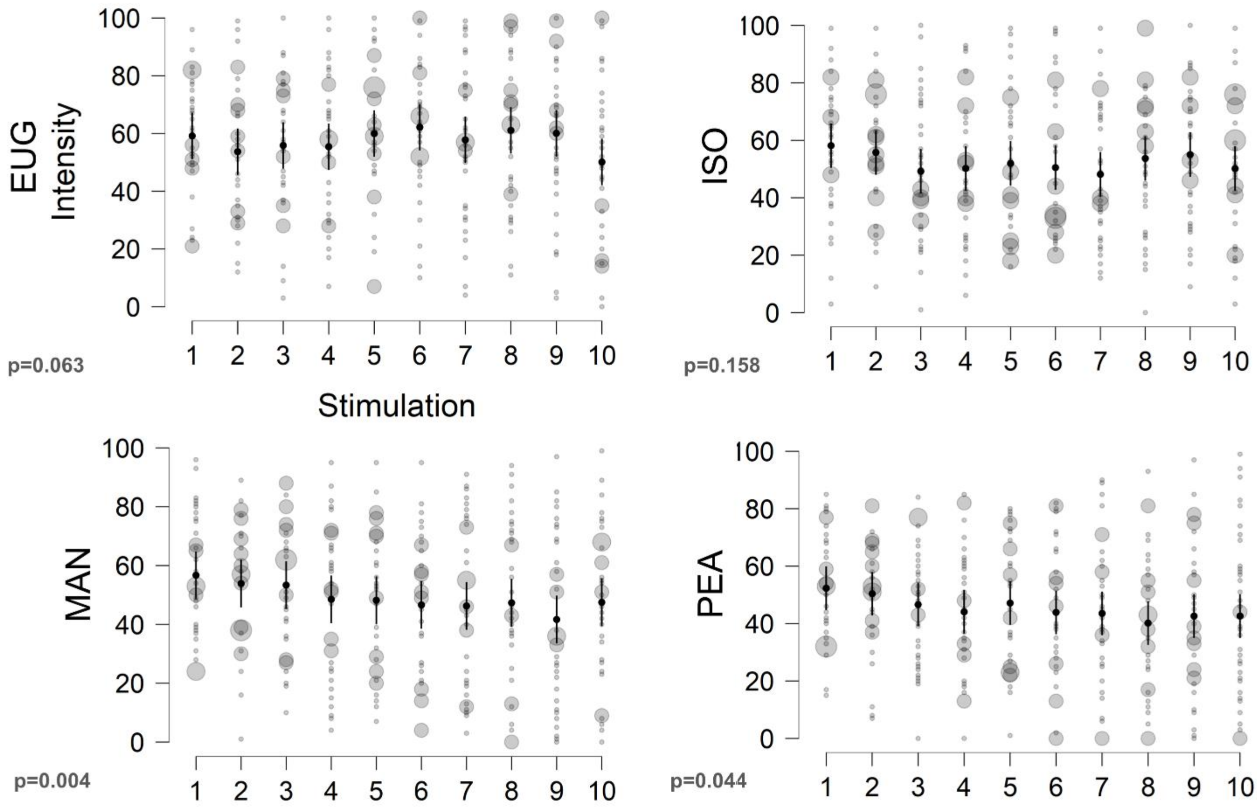


**Supplementary data 1: Individual intensity ratings over repeated stimulations for EUG, ISO E, MAN and PEA.** EUG = eugenol, ISO = Iso E Super, MAN = manzanate, PEA = phenyl-ethyl alcohol. Grey dots represent individual intensity ratings, with dot size corresponding to the number of subjects giving the same rating. Black dots correspond to the mean intensity rating at each trigger. Black bars represent CI95%.

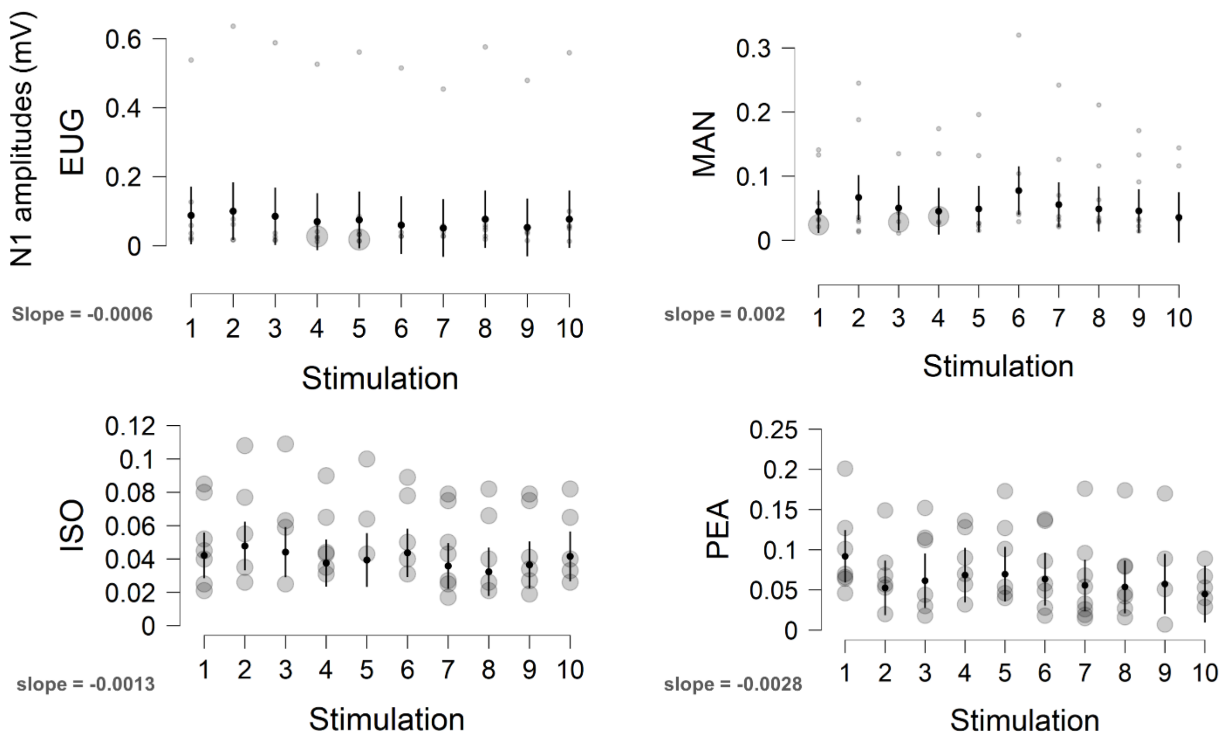


**Supplementary data 2: Individual N1 amplitudes over the stimulations with the four odors. EUG = eugenol, ISO = Iso E Super, MAN = manzanate, PEA = phenyl-ethyl alcohol.** Grey dots represent individual N1 amplitudes, with dot size corresponding to the number of subjects having quite the same amplitude. Black dots correspond to the mean N1 amplitude at each trigger. Black bars represent CI95%.
